# Acute arterial occlusion resulting from paradoxical embolism – case report and literature review

**DOI:** 10.1590/1677-5449.210074

**Published:** 2021-12-13

**Authors:** Juliana Daud Ribeiro, Marcio Barreto de Araujo, Barbara de Araujo Casa, João Antonio Correa

**Affiliations:** 1 Centro Universitário FMABC, Santo André, SP, Brasil.

**Keywords:** embolia, embolia paradoxal, doença arterial periférica, forame oval pérvio, trombose venosa profunda, embolia pulmonar

## Abstract

A embolia paradoxal é a transposição de um trombo originário da circulação sistêmica venosa para a arterial através de um defeito cardíaco, mais comumente o forame oval pérvio (FOP). A manifestação mais comum é o acidente cerebrovascular. A oclusão arterial aguda (OAA) é rara, requer alta suspeição diagnóstica e corresponde a menos de 2% de todos casos de embolia arterial. O tromboembolismo pulmonar (TEP) é a causa mais comum de elevação temporária do *shunt* direita-esquerda em pacientes com FOP e ocorre em pelo menos 60% dos casos de embolia paradoxal. Em 2019, um homem de 27 anos, sem fator para hipercoagulabilidade, deu entrada no Hospital Universitário do ABC, com quadro de OAA grau I Rutherford em membros inferiores secundária a tromboembolismo através de FOP prévio não diagnosticado, associado a trombose venosa profunda de membro inferior direito e TEP bilateral. O manejo incluiu anticoagulação plena e encaminhamento para cirurgia cardíaca.

## INTRODUCTION

Patent foramen ovale (PFO) was first described in 1877 by Cohnheim, who followed the path of an embolus through a septal defect in the heart. A PFO is a communication between the atria that is common in fetal life but at birth, in response to the first breaths, the PFO closes because left atrium pressure becomes greater than right atrium pressure. A persistent foramen that can potentially be crossed by a thromboembolism is a defect with an estimated prevalence of 15-35% of the adult population, according to cadaveric studies.^1^^,^^2^^,^^3^

The most common consequence of paradoxical embolism is stroke, while acute arterial occlusion (AAO) is rare. We report the case of a 27-year-old male patient with Rutherford grade I AAO caused by thromboembolism of the common femoral artery, femoral artery, right deep femoral artery, and left popliteal artery caused by an undiagnosed PFO. The objective of this article is to aid in the practicalities of identification and management of this disease.

The research protocol was approved by the Ethics Committee at our institution (decision number 4.709.780, CAAE 45679320.8.0000.0082).

## CASE REPORT

A 27-year-old male patient with no comorbidities other than grade II obesity (body mass index: 36.2) was admitted to our service for treatment of AAO of the left lower limb, found during investigation to test for deep venous thrombosis (DVT) after an episode of bilateral pulmonary thromboembolism (PTE). The patient presented with discrete lower limb pain, absence of motor or sensory deficits, absence of temperature gradient, absence of posterior tibial pulses bilaterally, absence of anterior tibial and popliteal pulses in the left lower limb, although with sounds audible on Doppler, and the remaining right lower limb pulses weak, which classified his condition as Rutherford grade I acute ischemia.

Investigations conducted with a range of equipment was positive for dyspnea in response to minor effort and significant pain and edema in the lower limbs that worsened when standing. The patient stated he had no history of venous or pulmonary thromboembolism or other types of cardiac conditions. He also denied smoking and other habits. His family history was negative for hematological diseases. Of 12 siblings, two had died in childhood from unidentified natural causes, while the remainder had no comorbidities. His parents were hypertensive and his maternal grandmother had died from decompensated heart failure.

The patient reported that he had recently traveled by bus, making a journey lasting three days each way, staying 1 month between outward and return legs of the trip. His symptoms had started on the second day of the trip, with claudication involving the left lower limb in response to minor effort, but he had not sought medical attention while away. Dyspnea had compounded his condition 4 days after his return. Claudication involving the right lower limb had started on the eighth day, by when he was no longer able to walk up stairs and the toes of both feet were cold, while the lower limb pain and dyspnea both improved when at rest. During the night he suffered an episode of syncope and the following morning a relative had taken him to an emergency walk-in center from where he had been transferred to the walk-in center’s referral hospital.

**Figure 1:**
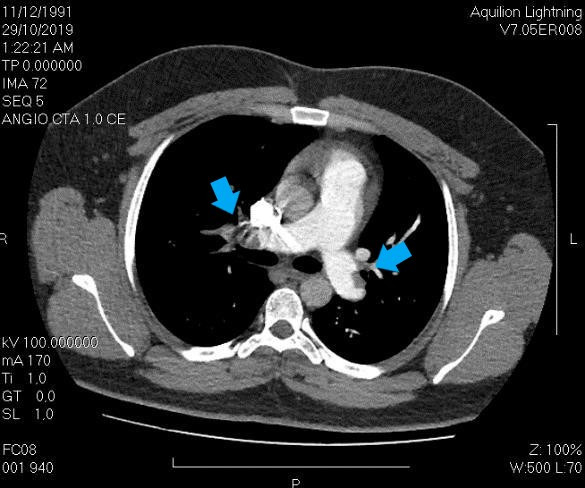
Pulmonary thromboembolism tomography protocol, positive for bilateral pulmonary thromboembolism (blue arrows).

The patient was admitted to the hospital with tachycardia and dyspnea, with electrocardiogram evidence of tachycardia, signs of right ventricle overload, and anterior T-wave inversion. A transthoracic echocardiogram also showed evidence of dysfunction of the right chambers, discrete movement of the interventricular septum, and indirect signs of pulmonary hypertension, but images suggestive of thrombus were absent; while tomography was positive for bilateral PTE (Figure 1). He was put on full anticoagulation and an ultrasound examination of the left lower limb venous system was conducted.

Laboratory tests did not detect abnormalities, with creatine phosphokinase at 90 U/L. Doppler ultrasound of the left lower limb venous system did not detect deep venous thrombosis (DVT), but a hypoechoic image was seen in the region of the popliteal artery, at the height of the joint line, with flow absent using color and spectral modes, compatible with popliteal occlusion. After this finding, the patient was transported to the vascular surgery department at our service.

**Figure 2:**
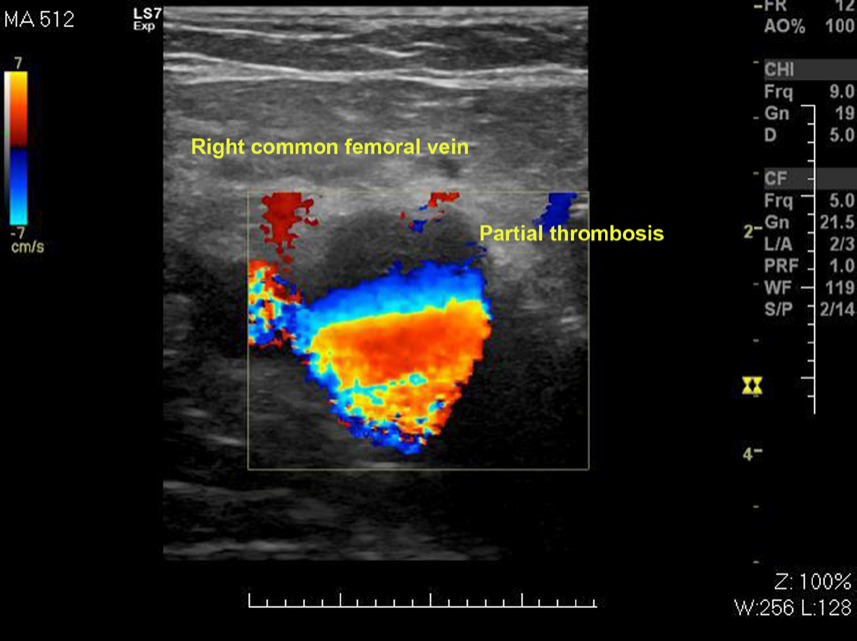
Subacute partial venous thrombosis of the common femoral vein.

**Figure 3:**
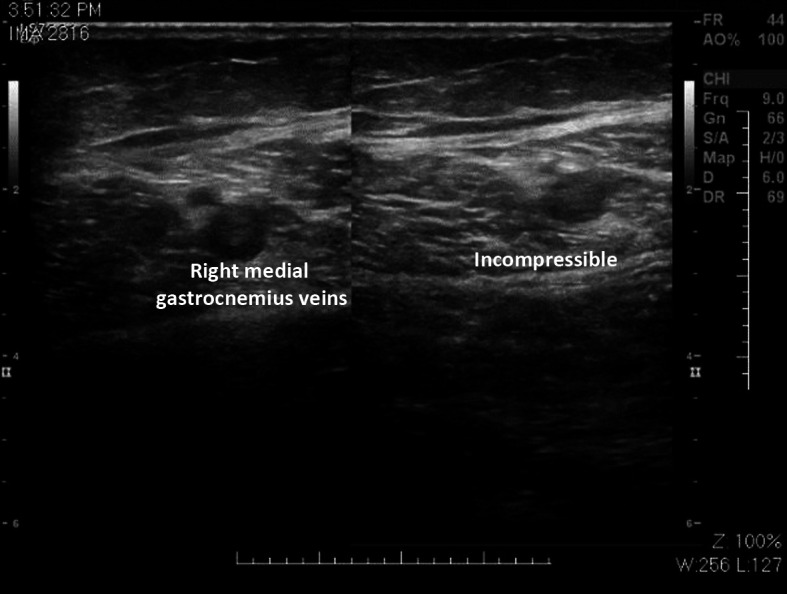
Subacute venous thrombosis of gastrocnemius veins.

**Figure 4:**
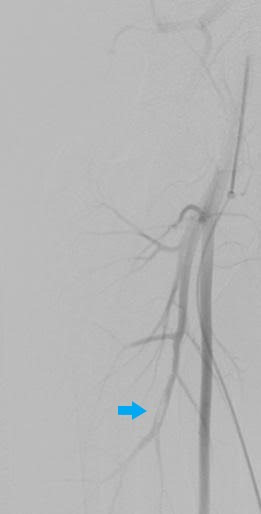
Digital subtraction angiography photograph showing the deep femoral artery with image suggestive of thrombus in one of its branches (blue arrow).

**Figure 5:**
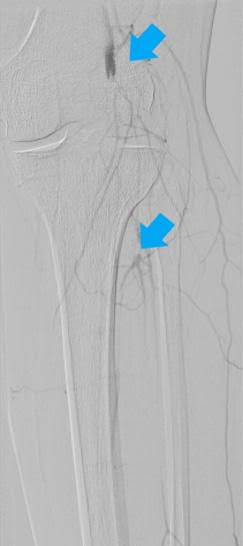
Digital subtraction angiography photograph demonstrating occlusion of the suprapatellar segment of the popliteal artery with no re-filling (upper blue arrow) and tibioperoneal trunk with re-filling close to the bifurcation (lower blue arrow).

**Figure 6:**
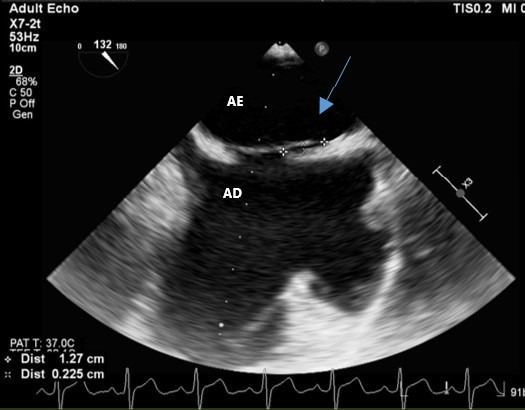
Patent foramen ovale (blue arrow). AD: right atrium; AE: left atrium.

At our institution, a supplementary investigation was conducted consisting of the following procedures: 1) Doppler ultrasound of the right lower limb deep venous system, which found subacute venous partial thrombosis of the common femoral vein (Figure 2) and gastrocnemius veins (Figure 3); 2) pre-angiography Doppler ultrasound of lower limb arteries; 3) aortography, with no abnormal findings; 4) arteriography of the right lower limb, showing the deep femoral artery with images suggestive of thrombus in one of its branches (Figure 4), popliteal and infrapatellar arteries with parietal irregularities, anterior tibial artery occluded at the transition to the dorsalis pedis artery, and fibular artery occluded at the middle third, with no filling, and incomplete plantar arch; 5) arteriography of the left lower limb, showing the suprapatellar segment of the popliteal artery occluded, with no re-filling, tibioperoneal trunk with re-filling soon before its bifurcation (Figure 5), anterior tibial artery and posterior tibial artery occluded at their origins, with no re-filling, fibular artery patent, with stenosis less than 50% and parietal irregularities, and plantar arch with no contrast uptake; 6) transesophageal echocardiogram, showing PFO (margins of the foramen > 5 mm), interatrial septum with 0.25 mm detachment from the fossa ovalis and a 13 mm long tunnel, with absence of thrombi or vegetations (Figure 6); and 7) thrombophilia tests, which were negative.

The patient was put on conservative treatment with full anticoagulation with enoxaparin while in hospital. At the end of his hospital stay, the patient had improved, with strong pulses present in the right lower limb, a right ankle-brachial index > 1.0, and with weak popliteal pulse returning in the left lower limb, although the left distal pulses remained absent and the left ankle-brachial index was 0.53. He was discharged from hospital on edoxaban 60 mg 1x/day with a referral for outpatients scheduling of repair of the PFO with heart surgery. No follow-up imaging exams were performed because of the COVID-19 pandemic.

## DISCUSSION

No other similar reports were found in the Brazilian literature. In a study conducted within the interventional cardiology program at the University of California, 416 patients were referred for PFO closure from 2001 to 2009. Just 4 (0.96%) of them had peripheral arterial emboli, one in a popliteal artery, two in ophthalmic arteries, and one in a brachial artery.^4^

A retrospective study covering the period from 1989 to 1999 reported that paradoxical embolisms were responsible for 13 cases of AAO. At a large vascular surgery service, it would be expected that at least one patient per year would be diagnosed with this etiology of AAO.^5^

For paradoxical embolism to occur, the shunt must occur at the specific moment when the thrombus is inside the right atrium, since it is not sufficient that the patient has a PFO – a right-left shunt via the foramen must occur. A right-left shunt may occur with increased volume or intrathoracic pressure.^6^

A study of the prevalence of DVT in patients with suspected paradoxical embolism concluded that when a PFO was detected in patients with embolism, there was frequently occult venous thrombosis in lower limbs. The same study examined 264 patients with suspected embolic events using transesophageal echocardiogram. Forty-nine of these patients (24 women and 25 men) had a PFO, 41 of whom suffered strokes and 8 of whom had AAO.^7^

In another study, the authors questioned whether paradoxical embolism and AAO were rare events or were suspected rarely and suggested that patients with AAO should undergo angiography and if no embolic source was found, should then undergo transesophageal echocardiogram. If a PFO with right-left shunt was diagnosed and there was evidence of DVT or paradoxical embolism, the PFO should be closed. If the evidence was negative, the PFO should be closed in cases with significant or recurrent events, and vena cava filters and/or warfarin would be recommended for 12 months, followed by antiplatelet therapy (Recommendation Grade C).^8^^,^^9^^,^^10^

The mean age of patients with paradoxical embolism secondary to PFO is 54 years, affecting men and women equally. The majority involve the lower limbs, primarily the left lower limb. Cases in which multiple limbs are involved are rare and concomitant DVT and PTE are common. Initial management should be the same as for other AAO. The majority of patients need urgent thromboembolectomy and/or fibrinolysis, with or without fasciotomy, and amputation is rarely needed.^1^^,^^11^

Investigation includes electrocardiogram, transthoracic or transesophageal echocardiogram, Doppler ultrasound of the lower limb deep venous system if the clinical picture is suggestive of PFO or PFO is identified, and tests for thrombophilias (Recommendation Grade C).^1^^,^^12^^,^^13^

In the case reported, surgical treatment was not needed and multiple limbs were involved, which is seen in a minority of cases in the literature. The patient in question was also young in relation to the age group most often affected. The fact that within a 1-month period the patient had undertaken two journeys lasting 3 days each in a bus with few stops and little mobility, was an important factor predisposing to formation of thrombus in the venous circulation, and, since there were no other more probable etiologies, was a crucial factor in suspicion of paradoxical embolism.

The period on anticoagulation proposed by our team was 6 months or until the PFO was closed. There are no predictive factors to identify or quantify the risk of paradoxical embolism in previously diagnosed shunts.

In more than 80% of cases, embolism is a consequence of cardiac thromboembolism. However, in patients in whom AAO has no defined etiology, differential diagnosis with paradoxical embolism secondary to PFO should be considered 1^,^^13^^,^^14^^,^^15^. The intention of this article is to contribute to expanding knowledge on the subject and to increasing suspicion of this rare disease.
