# Supplementary material for: Acute arterial occlusion resulting from paradoxical embolism – case report and literature review
Source: J Vasc Bras. 2021 Dec 13;20:e20210074. doi: 10.1590/1677-5449.210074 (PMC8719454; doi:10.1590/1677-5449.210074)
Supplement: Translation

# Translation: Oclusão arterial aguda consequente a embolia paradoxal – relato de caso e revisão de literatura

**Authors:** Juliana Daud Ribeiro, Marcio Barreto de Araujo, Barbara de Araujo Casa, João Antonio Correa

**Affiliations:** 1 Centro Universitário FMABC, Santo André, SP, Brasil.

**Keywords:** embolia, embolia paradoxal, doença arterial periférica, forame oval pérvio, trombose venosa profunda, embolia pulmonar

## Abstract

A embolia paradoxal é a transposição de um trombo originário da circulação sistêmica venosa para a arterial através de um defeito cardíaco, mais comumente o forame oval pérvio (FOP). A manifestação mais comum é o acidente cerebrovascular. A oclusão arterial aguda (OAA) é rara, requer alta suspeição diagnóstica e corresponde a menos de 2% de todos casos de embolia arterial. O tromboembolismo pulmonar (TEP) é a causa mais comum de elevação temporária do *shunt* direita-esquerda em pacientes com FOP e ocorre em pelo menos 60% dos casos de embolia paradoxal. Em 2019, um homem de 27 anos, sem fator para hipercoagulabilidade, deu entrada no Hospital Universitário do ABC, com quadro de OAA grau I Rutherford em membros inferiores secundária a tromboembolismo através de FOP prévio não diagnosticado, associado a trombose venosa profunda de membro inferior direito e TEP bilateral. O manejo incluiu anticoagulação plena e encaminhamento para cirurgia cardíaca.

## INTRODUÇÃO

O forame oval pérvio (FOP) foi primeiramente descrito por Cohnheim, em 1877, que traçou o caminho de um êmbolo através de um defeito septal no coração. O FOP é uma comunicação entre os átrios comum na vida fetal, porém, ao nascimento e à primeira respiração, o FOP se fecha, pois a pressão do átrio esquerdo se torna maior do que a do átrio direito. A persistência desse forame, potencialmente atravessável por um tromboembolismo, é um defeito estimado em 15-35% da população adulta, segundo estudos em cadáveres^1^^,^^2^^,^^3^.

Acidentes cerebrovasculares são a consequência mais comum de embolia paradoxal, sendo rara a oclusão arterial aguda (OAA). Relatamos o caso de um paciente de 27 anos com OAA Rutherford Classe I por um tromboembolismo em artéria femoral comum, artéria femoral, artéria femoral profunda direita e artéria poplítea esquerda devido a um FOP não diagnosticado. O objetivo deste artigo foi auxiliar na praticidade da identificação e manejo dessa doença.

O protocolo foi aprovado pelo Comitê de Ética de nossa instituição (parecer número 4.709.780, CAAE 45679320.8.0000.0082).

## RELATO DE CASO

O paciente de 27 anos, masculino e sem comorbidades, exceto por obesidade grau II (índice de massa corporal 36,2), deu entrada em nosso serviço para o tratamento de OAA de membro inferior esquerdo, achado em exame para pesquisa de trombose venosa profunda (TVP) após episódio de tromboembolismo pulmonar bilateral (TEP). O paciente apresentava discreta dor em membros inferiores, ausência de déficit motor ou sensitivo, ausência de gradiente térmico, ausência de pulsos tibiais posteriores bilateralmente, ausência de pulso tibial anterior e poplíteo em membro inferior esquerdo, porém com som audível ao Doppler, e demais pulsos em membro inferior direito diminuídos, o que o classificou como Rutherford Classe I de isquemia aguda.

O interrogatório sobre os diversos aparelhos foi positivo para dispneia aos pequenos esforços, dor e edema importante nos membros inferiores, com piora ao ficar em pé. O paciente negou histórico de tromboembolismo venoso ou pulmonar ou demais moléstias cardíacas. Negou tabagismo ou vícios. A história familiar foi negativa para doença hematológica. Entre 12 irmãos, dois morreram na infância de morte natural não esclarecida, e os demais não apresentaram comorbidades. Os pais eram hipertensos, e a avó materna faleceu por insuficiência cardíaca descompensada.

O paciente referiu viagem recente de ônibus, com duração de 3 dias na ida e na volta e tempo de permanência de 1 mês. Os sintomas iniciaram-se desde o segundo dia de viagem, com claudicação em membro inferior esquerdo aos pequenos esforços, porém não procurou assistência médica durante a estadia. Quatro dias após o retorno, iniciou dispneia associado ao quadro. No oitavo dia, apresentou claudicação no membro inferior direito, não tolerava subir escadas, apresentava frialdade em pododáctilos bilateralmente e melhora da dor em membros inferiores e da dispneia ao repouso. Pela noite, apresentou episódio de síncope, e, na manhã seguinte, foi levado a Unidade de Pronto Atendimento (UPA) por um familiar, tendo sido, após, transferido ao Hospital referência da UPA.

Nesse hospital, o paciente foi admitido taquicárdico e dispneico, com eletrocardiograma evidenciando taquicardia, sinais de sobrecarga de ventrículo direito e inversão de onda T em parede anterior. Também apresentou ecocardiograma transtorácico evidenciando sinais de disfunção das cavidades direitas, discreta movimentação paradoxal do septo interventricular, sinais indiretos de hipertensão pulmonar e ausência de imagem sugestiva de trombo; além de tomografia positiva para TEP bilateral (Figura 1). Foram realizados anticoagulação plena e ultrassom venoso de membro inferior esquerdo.

**Figura 1:** Tomografia protocolo T.E.P., positiva para tromboembolismo pulmonar bilateral (setas azuis).

Os exames laboratoriais não evidenciaram alterações, incluindo creatinofosfoquinase de 90 U/L. O ultrassom Doppler venoso do membro inferior esquerdo não evidenciou TVP, porém observou-se imagem hipoecoica em topografia de artéria poplítea, na altura da interlinha articular, com ausência de fluxo tanto ao método color quanto à análise espectral, compatível com oclusão da mesma. Após esse achado, o paciente foi encaminhado para a avaliação da equipe de Cirurgia Vascular em nosso serviço.

Em nossa instituição, foi realizado o estudo complementar com os seguintes procedimentos: 1) ultrassom Doppler venoso profundo de membro inferior direito, evidenciando trombose venosa subaguda de veia femoral comum parcialmente (Figura 2) e de veias gastrocnêmias (Figura 3); 2) ultrassom Doppler arterial de membros inferiores pré-angiografia; 3) aortografia sem alterações; 4) arteriografia do membro inferior direito, que mostrou artéria femoral profunda com imagem sugestiva de trombo em um de seus ramos (Figura 4), artéria poplítea e infrapatelares com irregularidades parietais, artéria tibial anterior ocluída na transição com a artéria pediosa e artéria fibular ocluída em terço médio sem reenchimento, com arco plantar incompleto; 5) arteriografia de membro inferior esquerdo, com artéria poplítea ocluída em segmento suprapatelar sem reenchimento, evidenciando tronco tibiofibular com reenchimento logo antes da sua bifurcação (Figura 5), artéria tibial anterior e artéria tibial posterior ocluídas na origem sem reenchimento, artéria fibular pérvia, com estenoses menores que 50% e irregularidades parietais e arco plantar não contrastado; 6) ecocardiograma transesofágico, com FOP (bordas do forame > 5 mm), septo interatrial com descolamento da fossa oval de 0,25 mm e túnel longo de 13 mm e ausência de trombos ou vegetações (Figura 6); e 7) provas de trombofilia negativas.

**Figura 2:** Trombose venosa subaguda de veia femoral comum parcialmente.

**Figura 3:** Trombose venosa subaguda de veias gastrocnêmias.

**Figura 4:** Fotografia de angiografia com subtração digital que demonstra artéria femoral profunda com imagem sugestiva de trombo em um de seus ramos (seta azul).

**Figura 5:** Fotografia de angiografia com subtração digital que demonstra artéria poplítea ocluída em segmento suprapatelar sem reenchimento (seta azul superior) e tronco tibiofibular com reenchimento logo antes da sua bifurcação (seta azul inferior).

**Figura 6:** Forame oval pérvio (seta azul). AD: átrio direito; AE: átrio esquerdo.

Foi instituído o tratamento conservador com anticoagulação plena com enoxaparina durante a internação. Após o período de internação, o paciente evoluiu com pulsos presentes e cheios em membro inferior direito com índice tornozelo-braquial (ITB) > 1,0 e com surgimento de pulso poplíteo diminuído em membro inferior esquerdo, apesar de manter a ausência de pulsos distais, com ITB de 0,53. O paciente teve alta hospitalar com edoxabana 60 mg 1x/dia e encaminhamento para programação ambulatorial de correção do FOP com a cirurgia cardíaca. Não foram feitos exames de imagem no seguimento devido à pandemia de covid-19.

## DISCUSSÃO

Não foi encontrado outro relato semelhante na literatura nacional. Em um estudo no programa de cardiologia intervencionista da Universidade da Califórnia, 416 pacientes foram referenciados, entre 2001 e 2009, para o fechamento do FOP. Destes, apenas 4 (0,96%) apresentaram embolia arterial periférica, sendo um para a artéria poplítea, dois para a artéria oftálmica e um para a artéria braquial^4^.

Entre 1989 e 1999, em um estudo retrospectivo, a embolia paradoxal foi responsável por 13 casos de OAA. Em um serviço grande de Cirurgia Vascular seria esperado que pelo menos um paciente por ano fosse diagnosticado com essa etiologia para OAA^5^.

Para que ocorra a embolia paradoxal, o *shunt* deve ocorrer no momento específico em que o trombo se encontre no átrio direito, visto que não basta o paciente ter o FOP – deve ocorrer o *shunt* direita-esquerda pelo forame. O *shunt* direita-esquerda pode ocorrer com o aumento do volume ou da pressão intratorácica^6^.

Um estudo sobre a prevalência de TVP em pacientes com suspeita de embolia paradoxal concluiu que, quando o FOP foi detectado nos pacientes com embolismo, foi frequente a presença de trombose venosa oculta em membros inferiores. Nesse mesmo estudo, 264 pacientes com suspeita de eventos embólicos foram submetidos ao ecocardiograma transesofágico. Destes, 49 pacientes (24 mulheres e 25 homens) tinham FOP, dos quais 41 apresentaram acidente vascular cerebral e 8 tiveram OAA^7^.

Em outro estudo, os autores questionaram se a embolia paradoxal e a OAA eram eventos raros ou pouco suspeitados e sugeriram que pacientes com OAA devessem ser submetidos a angiografia e, caso não houvesse fonte embólica, fossem submetidos ao ecocardiograma transesofágico. Caso fosse diagnosticado o FOP com *shunt* direita-esquerda e houvesse evidência de TVP ou embolia paradoxal, deveria ser realizada a correção do FOP. Caso a evidência fosse negativa, o FOP deveria ser fechado nos casos de eventos significativos ou recorrentes, e o filtro de veia cava e/ou a varfarina seriam as terapias preconizadas ao longo de 12 meses, seguidas de regime antiplaquetário (Grau de Recomendação C)^8^^,^^9^^,^^10^.

A idade média de pacientes com embolia paradoxal secundária a FOP é de 54 anos, acometendo igualmente homens e mulheres. Em sua maioria, acomete os membros inferiores, principalmente o membro inferior esquerdo. Os casos com múltiplos membros afetados são infrequentes, e TVP e TEP são comumente associados. O manejo inicial deve ser igual ao de outras OAA. A maioria dos pacientes necessita ser submetida a tromboembolectomia de urgência e/ou fibrinólise, com ou sem fasciotomia, e a amputação raramente ocorre^1^^,^^11^.

Os exames complementares incluem eletrocardiograma, ecocardiograma transtorácico ou transesofágico, ultrassom Doppler venoso profundo de membros inferiores caso haja clínica sugestiva ou após a identificação de FOP e investigação de trombofilias (Grau de Recomendação C)^1^^,^^12^^,^^13^.

No caso apresentado, não foi necessária abordagem cirúrgica e houve acometimento de múltiplos membros, correspondendo a uma minoria em relação à literatura, como também o paciente em questão era jovem em relação à faixa etária mais acometida. O fato de uma viagem com duração de 3 dias ter sido realizada duas vezes em um período de 1 mês, dentro de um ônibus com poucas paradas e pouca mobilidade, foi um fator importante para predispor a formação de trombo na circulação venosa, e, por não haver outras etiologias mais prováveis, foi fator crucial para a suspeita de embolia paradoxal.

O tempo de anticoagulação proposto pela nossa equipe foi de 6 meses ou até a correção do FOP. Não há fatores preditivos para identificar ou quantificar o risco de embolia paradoxal em *shunts* cardíacos previamente diagnosticados.

A embolia é, em mais de 80% dos casos, consequência do tromboembolismo cardíaco. Porém, em pacientes em que a OAA não tem etiologia definida, o diagnóstico diferencial com embolia paradoxal secundário a FOP deve ser considerado^1^^,^^13^^,^^14^^,^^15^. Este artigo tem o intuito de contribuir e expandir o conhecimento sobre o assunto, bem como aumentar a suspeição dessa rara doença.
